# Dental calculus in the industrial age: Human dental calculus in the Post-Medieval period, a case study from industrial Manchester

**DOI:** 10.1016/j.quaint.2021.09.020

**Published:** 2023-04-20

**Authors:** Lisa MacKenzie, Camilla F. Speller, Malin Holst, Katie Keefe, Anita Radini

**Affiliations:** a Department of Archaeology, University of York, York, YO1 7EP, UK, YO1 UK; b Department of Anthropology, University of British Columbia, Vancouver, V6T 1Z1, Canada; c York Osteoarchaeology Ltd., Bishop Wilton, York, YO42 1SR, UK; d York JEOL Nanocentre, University of York, YO10 5DD, UK

**Keywords:** Bioarchaeology, Dental calculus microscopy, Post-medieval period, Food hygiene, Food processing, Imported species

## Abstract

The analysis of dental calculus (mineralised dental plaque) has become an increasingly important facet of bioarchaeological research. Although microscopic analysis of microdebris entrapped within dental calculus has revealed important insights into the diet, health, and environment of multiple prehistoric populations, relatively few studies have examined the contributions of this approach to more recent historical periods. In this study, we analyze dental calculus from an English Post-Medieval, middle-class urban skeletal assemblage from Manchester, England using light microscopy. We characterize all types of microremains, observing heavily damaged starch and plant material, high quantities of fungal and yeast spores, the presence of wood particles, plant (cotton) and animal (wool) fibres, as well as limited quantities of microcharcoal and burnt debris. We observe the presence of non-native, imported plant products, including New World maize and potentially tapioca starch. We compare our results to similar studies from earlier time periods to reveal the impacts of the significant economic, social and environmental changes occurring during the Industrial period in England, including changes in food processing, food access, food storage, and air quality. We conclude by outlining important methodological considerations for the future study of Post-Medieval dental calculus and propose potential areas of future research.

## 1 Introduction and aims of this paper

### 1.1 Industrial Revolution and dental calculus

What do we think of when we picture the Industrial Revolution (ca. 1750–1850) in the North of England?: dark, smoke-filled cities; Dickensian tales of the squalid conditions of the poor and their exploitation in factories. For many, however, the reality of the Industrial Revolution was that it was a time of innovation, improvement of housing, transport, culture and learning. These changes were instigated and experienced by the newly emerging middle-classes, who during the Enlightenment Period (1715–1789) continued to revolutionise science, engineering and medicine, aspiring to create a better society for all. Archaeologically, this period is both important and tangible. Many buildings of the period are still in use today as are many of the standards for industry which were being explored and developed in the eighteenth century: the beginnings of mass production, larger industry, lowering costs, whilst transitioning production from primarily workshops to factories. The eighteenth century was also a time when middle-class consumers were focused heavily on novelty (new products and foods), convenience (indoor emporiums versus open marketplace), and the consumption of exotic goods brought from the far reaches of the Empire, the latter practice which served to emphasise their social status and good taste (Stobart, 2012; Hann and Stobart, 2005). In this study, we consider how the emerging middle-class experienced this transition towards convenient, ready-made, and potentially mass-produced goods versus artisanal hand-produced goods (i.e., food and drink) as well as changes in the methods of transport and storage of ‘prepared’ foodstuffs (i.e., flour). What impact did these new foods and forms of production have on the lived experiences of urban-dwelling middle-class individuals in the early stages of renewal in the north of England, specifically in Manchester?

Between 1750 and 1850, the population of England was growing rapidly, with higher numbers of people migrating from villages to cities, transitioning the country's economy from agricultural to industrial or capital-based (Hoppit, 1987, 213). This rural emigration primarily comprised young, working-age adults transferring the bulk of human capital to the cities (Williamson, 1988, 301). During the eighteenth century, road and canal networks were greatly expanded and improved, facilitating the transport of goods and people; in turn, these bolstered the transition from subsistence to market-oriented production, mitigating regional crop failures and famines (Hoppit, 1987, 213 l; Michaelowa, 2001, 10). Many of these advances were already in place before the introduction of steam-powered mechanisation and production, laying the groundwork for industrialisation in the latter half of the eighteenth century. Large urban centres have always been hubs for trade in the marketplace where people from surrounding areas could gather and share ideas. This amalgamation of people and ideas was further increased by expanding trade with imperial colonies which coincided with burgeoning industrialisation. Since the late seventeenth century, English shopping habits had begun to include ‘colonial groceries’ such as tea, coffee, and sugar (increasingly available in greater qualities as a result of slave labour), in addition to popular culinary spices such as pepper and nutmeg (Stobart, 2017, 1138).

Furthermore, amongst the hallmarks of industrialisation —advancements in technology, mechanisation and urbanisation— important social changes were also occurring, including the emergence of a middle-class, distinct from the working-classes and hereditary elites, which may differ from those of the medieval artisanal and merchant classes. This new middle-class rose from the ranks of the working-classes and were the forerunners of innovation and dissemination of new ideas and improvements in manufacturing, which would in turn mass-produce goods making them more affordable. This meant more buying, with more disposable income generated by reductions in food prices and increases in real wages (Lloyd-Jones and Roux, 1980; Barker, 2004). More goods meant more advertising, which further drove consumption habits, mainly in the middle- and working-classes (Skelton, 2003; Garrod, 2014). All this consumption and production would leave its mark on the environment as well, with more factories — many steam-powered— producing more air pollution, which many surmised had a detrimental effect on those who lived in affected areas (Bowler and Brimblecombe, 2000; Douglas et al., 2002). The city of Manchester, for example, was dubbed a ‘shock city’ due to its rapid expansion in area and influence on the manufacturing scene, and some suspect that much of this was unregulated by local authorities leading to deteriorating standards for food, housing and air quality (Kidd, 2002). In cities with a high concentration of factories, air quality was a major concern. Manchester was quick to regulate ‘nuisance smoke’ from factories and mills which had converted to coal and steam-driven machinery with legislation in the late eighteenth century (Bowler and Brimblecombe, 2000, 79). The well-documented London Fogs (smoke), beginning with the Great Fog of 1775, would plague the city during the winter months, and have been directly correlated with increased respiratory and circulatory diseases (Luckin, 2003, 33; Brickley et al., 2007; Pope and Dockery, 2006). The ‘fogs’ are thought to be exacerbated by coal hearths and cooking fires (Harrison and Aplin, 2002, 4043), which release polycyclic aromatic hydrocarbons (PAHs) from the incomplete combustion of coal, wood, oil, coke and coal tar which was most harmful in poorly ventilated kitchens and factories.

The north of England sat firmly at the pinnacle of industrial progress and innovation which makes it an interesting milieu to observe middle-class aspirations. In the century between 1750 and 1850, “Britain underwent extensive rebuilding and re-organisation in order to improve health, morality, cultural and civic life and the aesthetic experience of its wealthier inhabitants”, featuring clean streets (hidden sewers) free from human detritus (beggars and lunatics) and distasteful trades (butchery, tanning banished to outlying areas) to create a ‘moral’ landscape (Tarlow, 2007, 90 & 102–3). These improvements, however, were focused specifically upon the commercial districts and upper- and middle-class neighborhoods. The upper- and middle-class dwelling and commercial areas in Manchester were sharply delineated from working-class neighborhoods so that the misery and grime of the poor could be concealed from wealthy men and women (Engels, 1892: 46–47). Many of the eighteenth century middle-class improvements took the form of polite entertainments such as theatres, public gardens, and assembly rooms, as well as creating exclusive shopping districts with paved streets, public lighting, and extended evening shopping hours away from the open markets (Cox, 2000, 66–7). Closed shops allowed for the separation of the classes —“fertile spaces for polite social discourse”, especially creating a safe place of leisure for middle-class women to socialise amongst themselves (Wallis, 2008, 28). Nevertheless, in urban centres, virtually all households shifted from ‘market contact’ (purchasing goods to supplement household production) to a household economy focussed on wage labour, the most important source of income in north-western Europe in the eighteenth century (De Vries, 2008, 82). With more cash and fewer leisure days for making necessities at home, more people began to rely on purchased food and household goods. Closed shops began to proliferate and diversify to serve the needs of a more consumerist public.

Bioarchaeological methods applied directly to the skeletons of those who lived during these extraordinary technological, economic and social changes can provide us with novel information concerning the impacts of industrialisation on diet, health and the lived experience. Cemetery excavations have so far provided a wealth of physical information on Post-Medieval populations all over the United Kingdom, which has shed light on how mortality and morbidity have changed over time (Roberts and Cox, 2003) as well as morbidities associated with deteriorating dietary and living conditions worsening with increasing urbanisation, such as vitamin D deficiency (Brickley et al., 2007). An increased prevalence of skeletal and dental manifestations of malnutrition and chronic disease have been observed over this period (Crafts, 1997; Mant and Roberts, 2015; Roberts, 2016; Lewis, 2013), pointing to a quality of life lower than that of preceding periods, especially for the growing working-class populations, which experienced significantly reduced life expectancy compared to upper or middle-class individuals (Engels, 1892: 106–107; Lewis, 1999; Roberts and Cox, 2003: 303). Nevertheless, food access and other aspects of daily life, such as hygienic conditions and exposure to smoke and infectious diseases, can differentially affect individuals of different social classes, and may not be visible using osteoarchaeogical methods alone. Moreover, few studies have investigated the lived experience of the newly emerging middle-class in England using bioarchaeological methods (e.g., Brickley, 2006; Bleasdale et al., 2019). In the last few years, a mineralised deposit on teeth known as dental calculus has proven to be a reliable source of information on diet and living conditions in the past. Dental calculus results from the mineralisation of dental plaque (see section 2.1 of this paper for more details) and can entrap dietary and environmental particles and biomolecules that enter the human mouth, preserving them for millennia (Radini et al., 2017). These particles and molecules allow for the construction of integrated ‘*osteobiographies*’ of past individuals as never before, because they allow a direct link with the food and environment experienced by each individual during life. Despite its potential, dental calculus studies from individuals who lived in the Post-Medieval period are limited and confined to a small number of skeletons (e.g., Bleasdale et al., 2019; Geber et al., 2019).

### 1.2 General overview on dental calculus

Dental calculus is a hardened bacterial deposit on teeth resulting from the mineralisation of oral plaque (Lieverse, 1999). Plaque itself is a complex community of oral microbes which adhere to the surface of the teeth in increasing quantities and layers over time, creating a permeable environment through which sugars from saliva percolate prior to mineralisation (Hillson, 1996, 255). The transition to calculus occurs in two steps: 1) the plaque matrix becomes calcified; and 2) the bacteria themselves become mineralised via salivary calcium and phosphate ions forming crystals in conjunction with acidic phospholipids and proteolipids from the bacterial cell membranes increasing calcification (Clerehugh et al., 2013, 13).

During these transitions, particulate matter (for example, food and air-borne pollutants) and molecules present in the saliva can become entrapped in the mineralised matrix, a process which can be considered ‘fossilisation’ (Radini et al., 2017). The range of particulate matter extracted from calculus can vary exceedingly from one population to another and span from starch, phytoliths, pollen, fungal spores, insects remains, textile fibres and even pigments (e.g. Blatt et al., 2011; Gismondi et al., 2018; Radini et al., 2017; Radini et al., 2019).

Nevertheless, while calculus deposition rates are not well understood and deposits can represent an accumulation over a period of weeks, months or years (Lieverse, 1999), it is known that calculus formation ceases at death, ensuring that all the particles and molecules incorporated into the calculus matrix were encountered during the individual's lifetime. This aspect provides high archaeological integrity to all the entrapped materials (Radini et al., 2017). It has also become clear that the mineralised dental plaque is stable on dental enamel for millennia, as dental calculus deposits have been found on the teeth of a Miocene *Sivapithecus* dating to between 12 and 8 million years ago (Hershkovitz et al., 1997). Additionally, it has been shown in various biomolecular calculus studies that the hardened calcium carbonate matrix can effectively seal any ingested or inhaled particles passing through the mouth as well as preserving elements of the oral microbiome (symbiotic microbial community) and cellular structures of the individual (e.g., Metcalf et al., 2014; Warinner et al., 2014).

Due to its temporal breadth and the variety of information that can be retrieved from this deposit, dental calculus analysis is emerging as a new subfield of both osteoarchaeology, human palaeoecology and environmental archaeology. As stressed by Barton and Torrence (2015) the exciting aspect of a new research field is testing which new lines of inquiry emerge.

Even a brief survey of published data clearly shows that the initial focus of dental calculus research has been on dietary remains (Radini et al., 2017). However, in the past few years, the methodological and temporal research framework of ancient human dental calculus analysis has expanded (e.g., Gismondi et al., 2020; Radini et al., 2019; Tromp et al., 2017). Despite the expansion of research questions, systematic studies on Post-Medieval populations are generally lacking. The study presented here is part of recently completed Doctoral research aimed to address the lack of data for this period and to investigate how dental calculus research via light microscopy can inform our understanding of the social and economic complexities of the Industrial Revolution (MacKenzie, 2021).

### 1.3 Aims of the paper

This study quantifies and analyzes environmental and dietary debris observed in human dental calculus retrieved from individuals that lived and died in Manchester, England ca. 1700–1850. During this period, the City of Manchester and its population, witnessed some of the major technological and social changes dictated by an intense period of industrialisation and population growth, the impact of which is still felt today. Such a population therefore offers a unique opportunity to assess the contributions of dental calculus analysis when applied to the Post-Medieval period and in particular to the Industrial Revolution in an urban environment, further expanding our understanding of the research potential of this, often minute, dental deposit. In light of the general background provided above, it is clear that exploring the potential of dental calculus research in the Post-Medieval period can make a vital contribution to this emerging subfield. The aims of this paper are:

1. To survey the archaeological remains retrieved from the analysis of dental calculus samples from a middle-class urban population that lived and died during the Industrial period (ca. 1700–1850).
2. To assess how such remains may differ from those of the preceding time periods, and assess how they complement our understanding of the urban, middle-class lived experience during the Post-Medieval period.
3. To set a framework for future research in the analysis of Post-Medieval populations.

We hope that this paper will contribute to widening the field of research in human dental calculus, both methodologically and temporally.

## 2 Materials

### 2.1 Archaeological background

This study focuses on the skeletons recovered from the Cross Street Chapel cemetery, a non-conformist Unitarian chapel, located in the centre of Manchester, UK. It seems that the people buried in the Cross Street burial ground hailed from several affluent and cosmopolitan streets in the centre of Manchester, namely Princess Street, Ardwick, Mosley Street, Brazenose Street and Holme Street, suggesting “many members of the congregation of Cross Street were drawn from some of the more prestigious areas of the middle-class in Manchester” (CFA Archaeology Ltd, 2017b, 155–6). The Cross Street Chapel cemetery excavation was commissioned by Transport for Greater Manchester (project code TFGM3/2162) and conducted by CFA Archaeology Ltd. between September 2014 and November 2015, with the exhumation of 241 skeletons, 172 of which could be correlated with named individuals (CFA Archaeology Ltd, 2017a , 5–6). Excavations of the lower burial ground were located below the footpath and carriageway to the west of the chapel (due to road widening in the late nineteenth century) before the new Metrolink track was to be laid. The upper burial ground had been cleared of graves to build the Observatory office buildings in 1996, and the skeletons reburied without skeletal analysis. There was extensive disturbance caused by grave intercutting and intrusion from utility works since the late nineteenth century, which caused damage, disarticulation and partial loss of some skeletons, associated coffins and burial furnishings (CFA Archaeology Ltd, 2017a , 22).

Documentation of the grave markers and transcriptions had been conducted by Thomas Baker from 1854 to 1855 in a small book held by the Cross Street Chapel (CFA Archaeology Ltd, 2017a, 6–8, and 2017b, Appendix 3). A register of baptisms, marriages and deaths is held by the Manchester Central Library. The surviving burial register for Cross Street only covers the years 1785–1840, although burials took place from 1694 to 1852. After this the chapel continued as a place of worship until the destruction of the original chapel by bombing in the Second World War (1939–1945). The present chapel was built in 1997, partially covering some of the graves which were left in place (CFA Archaeology Ltd, 2017a , 53–5).

### 2.2 Osteoarchaeological information

A total of 36 individuals (16 males, 15 females, and 5 unsexed) were selected from the 241 recovered skeletons from the cemetery excavations. All of the selected individuals were adults aged between 18 and 46+ years of age with burial dates between 1737 and 1847 (CFA Archaeology Ltd, 2017b) (Supplementary Information Table S1). Standard aging techniques were applied (Scheuer and Black 2000a, 2000b; Cox, 2000), in particular those specific to the os-coxa (Brooks and Suchey, 1990; Lovejoy et al., 1985), and sternal rib ends (Iscan et al., 1984; Ubelaker, 1989). Sex estimation was based upon morphological traits observed in the pelvis and skull (Mays and Cox, 2000). All skeletal analyses were performed by Keefe and Holst (2017, 33 & 39) prior to sampling. Dental health was poor in the skeletal assemblage as a whole: high levels of tooth decay (caries) were observed (particularly affecting females), along with abscesses (particularly affecting males), and ante-mortem tooth loss (Supplementary Table S1).

**Fig. 1:**
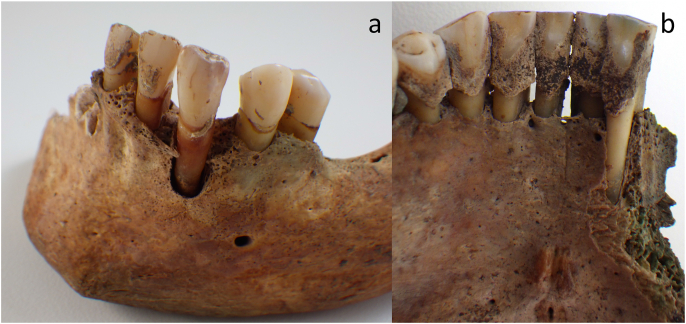
Examples of dental calculus from the Cross Street Chapel cemetery assemblage. A) SK4.04; b) SK4.36 (photos Malin Holst and Katie Keefe).

Selection criteria for the sample analysed here were based solely on the presence and amount of calculus preserved on the dentition, with those exhibiting larger deposits prioritised first (Fig. 1). Calculus deposits located in the region of major salivary glands (sublingual and parotid) located on the lingual surface of the lower incisors and the buccal surface of the maxillary molars were prioritised as these would have the greatest accumulation (Aghanashini et al., 2016). Overall, the calculus deposits were small and patchy (small flecks and flakes) with few large chunks, resulting in adjustments to the cleaning and extraction processes (see Methods).

## 3 Methods

### 3.1 Sampling and decontamination procedures

Calculus deposits were removed from the teeth using sterile dental scalers (cleaned with bleach between each individual) and deposited into sterile 2 mL Eppendorf tubes. Sterile powder-free double gloves were worn at all times and all calculus removal was conducted on sterile aluminium foil. Decontamination and extraction methods for microremains in human dental calculus analysis were adapted from those published by Cristiani et al. (2016) and Warinner et al. (2014) and successfully applied in other studies (e.g., Cristiani et al., 2018). Decontamination protocol followed two distinctive phases:

1. Phase 1. An initial removal of soil contaminants was performed by placing the calculus in an eppendorf tube, to which ultrapure water was added. The tube was then subjected to agitation and rotation until all visible surface burial soil and contaminants were removed (three cycles of agitation in ultra-pure water), following visual examination under a stereomicroscope.
2. Phase 2. Once all soil and debris were removed from the outer surface of the calculus by ultrapure water and agitation, the samples were inspected under a dissecting microscope. Any remaining contaminants (e.g., soil) were removed with the use of a sterile, ‘single use’, acupuncture needle and weak hydrochloric acid solution (0.6% HCl) before the cleaned dental calculus was immersed in HCl (0.6%) in a new Eppendorf tube and left to slowly dissolve over a period of days until the sample was completely decalcified.

During the decalcifying stage, dissolved calculus and surrounding natant (spent HCl) was pipetted onto sterile microscope slides, without additional mounting mediums (e.g., water and glycerol solutions) in order to accommodate any future chemical analyses. Mounting of calculus and natant on the slides was conducted in a designated ‘clean lab’, which is reserved solely for ancient materials and where contaminants are monitored, before being moved (in sealed containers) to the microscopy laboratory. Each slide was scanned in its entirety with high magnification (400×) using an inverted Olympus X71 microscope and all observed debris, both dietary and non-dietary, were recorded. This process was repeated until none of the calculus and natant were left in the tube. The scanning of the natant ensured that all microparticles released by the dissolving process of the dental calculus were retrieved. To ensure a sterile environment, dust test slides were left near all work in the clean lab as well as next to the microscope during slide scanning. There were no observed airborne contaminants for the duration of calculus analysis. Furthermore, as noted by Warinner et al. (2014) and Cristiani et al. (2016), these protocols allow the analyst to often observe microremains when they are still partially attached to calculus fragments and/or to calculus material in a ‘filamentous’ form —as seen in many of the pictures provided in this paper, and described by Warinner et al. (2014) as ‘pseudo-in situ’.

### 3.2 Identification and analysis of the remains

The identification of the remains was conducted with the use of an extensive ‘*purpose built*’ reference collection of microremains from plants, animals and crafts, consistent with species from Europe, North Africa and the Middle East used successfully in a wide number of collaborative works by the authors (e.g., Cristiani et al., 2018; Lucarini and Radini, 2020; Radini et al., 2016; Radini et al., 2019; Warinner et al., 2014), and currently located at the Laboratory of Microarchaeology at the University of York. The reference collection was complemented with economically important species of exotic plants that arrived in England during the Georgian period (1714–1837) via international trade. Modern plant material was processed (ground or cooked) during experimental work, reflecting changes in food processing which occurred after 1700. In addition to our extensive reference collection, a wide range of published work was also routinely consulted (e.g., Hassall, 1855; Accum, 1820; Winton and Moeller, 1906; Moss, 1976; Williams-Woodward, 2001). In order to make a meaningful comparison of microdebris composition between individuals and with other previously published research, all debris types were recalculated from their raw counts to parts-per-milligram of calculus for each individual (total count of debris type divided by calculus sample weight) before statistical analyses. This method is based on those used in populational dietary studies by Leonard et al. (2015) and Radini (2016) in order to observe broad population trends, since calculus accumulations are different for each individual.

### 3.3 Archive

All Cross Street individuals have been reinterred at Chorlton's Southern Cemetery near Manchester in January 2017 in the same family groups in which they were originally buried (BBC, 2015; Cox, 2017). Archives of the calculus material, analysed slides and reference collection are located at the University of York, UK in the Mary Cudworth Laboratory.

## 4 Results

### 4.1 General overview

The dental calculus analysis yielded a great variety of microremains, from all kingdoms of life as well as inorganic debris of potential cultural interest. The results are presented by kingdoms and summarized in Table 1. Due to the scattered nature of some remains, we have reported the key categories of remains in Table 1, while those retrieved from a very low number of individuals are only mentioned in the text, though their skeleton number is provided; all key finds are reported by skeleton in Supplementary Table S1. Criteria and reliability of identification are discussed by category below, with further work in progress or yet to be conducted noted for each category. Although the majority of the microremains were poorly preserved, it was possible to reach tribe and genus level identifications in some cases. Some types of remains were present in very large quantities, even when considering the very small amount of calculus available for analysis. The greatest number of remains were small fragments of undiagnostic plant matter (83%) and small fungal spores (6%), suggesting that the size of the particulate matter may influence the type of debris captured by the dental calculus matrix. The distribution of debris types was equal between the sexes, with the exception of soil and mineral grit which was more prevalent among females (4 females and 1 male), and wood debris which was slightly higher in males (12 males and 9 females). Nevertheless these number are low and, considering the very small size of dental calculus, care must be taken in interpreting differences in the concentration of debris between individuals (Supplementary Table S1).

**Table 1:** Percentage and total finds in the major debris categories for the Manchester sample group, broken down by sex. The debris categories are displayed by their raw counts (unadjusted for parts per milligram) with the median counts per sex, and with total and mean calculus weights.

	% of Total Finds	Raw count *n =*	Males (16)		Females (15)		Unsexed (5)	
			n =	Median	n =	Median	n =	Median
Plant Tissue	83	8255	4245	199	3168	219	865	192
Fungal Spores	6	594	247	7	334	3	13	2
Yeast Spores	4	382	77	2	284	8.5	14	6
Wood Particles	1	103	42	2.5	45	3	19	9.5
Starch Granules	0.5	55	23	1	28	2	4	2
Total Particles	94.5	9389	4634		3859		915	
Mean		1877.8	926.8		771.8		183	
Calculus in mg total			135.4		134.14		102.85	
Mean			8.4		8.94		20.57	

### 4.2 Kingdom Plantae

Plant micro-debris —ranging from starch granules to wood debris and fibres— was nearly ubiquitous among the sampled individuals but had clear evidence of damage. The majority of remains of plant origin were found in fragments too small to allow taxonomic identification, often below 5 μm in diameter. The remains are described below by their anatomical origin. Detailed information on the criteria for identification used are provided in Supplementary Table S2.

**Fig. 2:**
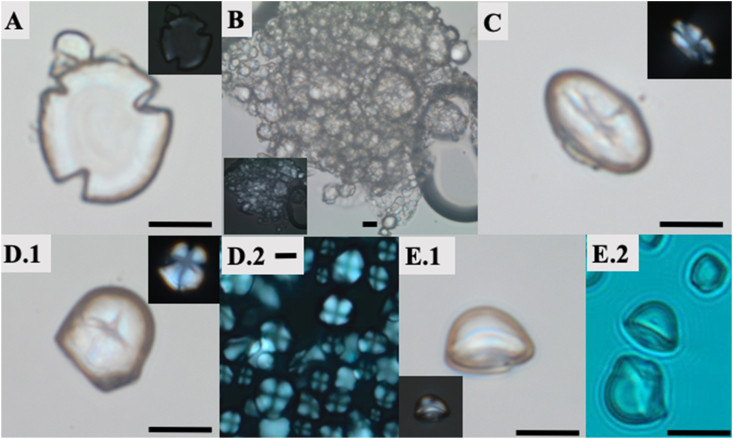
Ancient microremains from the analysed skeletons representing starch granules: A damaged starch granule, possibly bimodal Triticeae, displaying cracking and loss of birefringence (inset); B bimodal starch granules from the Triticeae tribe, observed clumped together within the calculus; C well preserved starch granule, likely legume (Fabaceae family); D.1 well preserved maize starch granule; D.2 modern example of *Zea maize* starch granules in our reference collection; E.1 a well preserved tapioca (*Manihot esculenta*) starch granule displaying diagnostic ‘kettle-drum’ three dimensional shape; E.2 *Manihot esculenta* starch granules from our modern reference collection. Scale bars 10 μm. Magnification 400×.

*Starch granules* were identified in 50% of the individuals examined, but the majority of the observed starch granules were too damaged to allow secure taxonomic identification (Fig. 2. A). Supplementary Table S2 presents the morphological criteria used for the identification and supporting published work. The starch granules that have clear morphological features were grouped in five morphotypes and belonged to the well-known staple crops of the Old and New World, with ample published record. Old World staple crops were represented by the starch Tribe Triticeae (wheat and barley- Fig. 2 B); the Fabeae (legumes- Fig. 2 C) and Tribe Aveneae (oats), while the New World crops were identified as imported species such as maize or Indian corn (*Zea maize-* Fig. 2 D) and tapioca (*Manihot esculenta-* Fig. 2 E). The damage observed in starch granules which prevented the identification of the majority of remains in this category is consistent with bloating and cracking and very likely occurred during processing and cooking —such changes are well known to cooking practice and well documented in the literature (e.g., Henry et al., 2009; Torrence and Barton, 2016). For the scope of this paper, we have reported all starch granules as a single group, and specified which individuals displayed imported species (See Table 1).

**Fig. 3:**
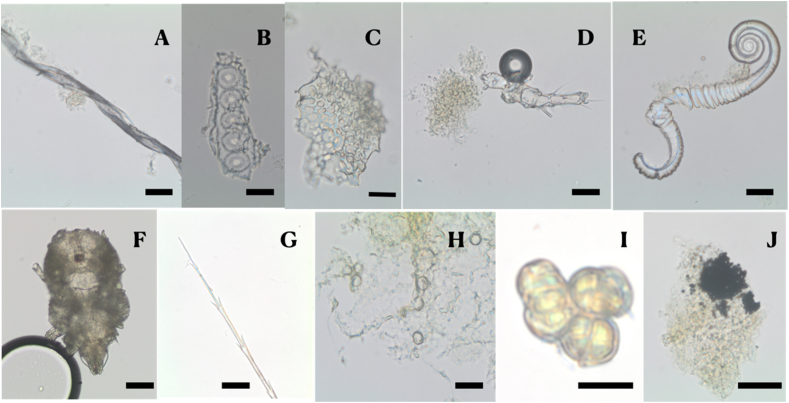
Ancient microremains from the analysed skeletons very likely representing non-food contaminants: A cotton (*Gossypium*) fibre, potentially dyed, with the distinctive ‘ribbon-like’ appearance; B fragment of coniferous softwood with characteristic pits, adhering flecks of calculus can also be seen; C an example of plant tissue, potentially a seed testa; D fragmented insect leg emerging from a calculus fleck; E well preserved but fragmented nematode with attached calculus; F nearly intact insect exoskeleton, possibly mite (Ascaris) encased in calculus, the head and one appendage are exposed (bottom of the image); G detail of a feather barbule (distal portion of the shaft); H yeast spores trapped in dental calculus, note the distinctive dimpled characteristic or ‘budding scar’; I potential *Spegazzinia* or *Ulocladium* type fungal spores; J minute particles of burnt material, possibly soot, incorporated into undissolved dental calculus. Scale bars 20 μm. Magnification 400×.

*Plant fibres (bast fibres and plant hairs)*, likely of textile origin, were recovered from 34 of the 36 (94%) Cross Street individuals (15 males, 15 females and 4 unsexed). Coarse fibres (>10 μm across) were identified as cotton (*Gossypium* spp.- Fig. 3 A) flax (*Linum usitatissimum*) and/or hemp (*Cannabis sativa*). Several finer fibres (<9 μm across) were also recovered; however, they were too small to observe secure identifiable features. We have provided the raw counts for these fibres separate from general plant fibres more likely coming from food in the event that future work will allow for their identification. Cotton fibres were identified by their ribbon-like appearance with flattened medulla, a smooth surface layer often with frayed fibre-ends and by comparison with our reference collection. While cotton has previously been identified in dental calculus from New World populations from Ohio (Blatt et al., 2011), to our knowledge, this is the first evidence of cotton to be recovered from Old World populations. Flax/hemp bast fibres were identified with cross-polarised light which revealed distinctive lumen (narrow central canal) with x-node dislocation scars (assists stem flexibility) and a distinct blue colouration (Kvavadze et al., 2010) and by comparison with the reference collection. The issues in the identification of flax versus hemp have been discussed in previous work (Warinner et al., 2014).

*Wood particles* were observed in 23 of the 36 (64%) of the individuals (12 males, 9 females and 2 unsexed) most often as very fine, partially digested particles with no identifiable features (undiagnostic), but a few originating from soft wood (coniferous trees such as pine trees - Pinaceae; Fig. 3 B) - were also recorded.

*Plant cells and tissues* were observed in all individuals, consisting of single or clustered cellular structures identifiable as plants by the thick cellular walls of the particles (Fig. 3 C). The taxonomic identification of such remains is however problematic due to poor status of preservation. Pollen was found in only one individual (SK 1.14).

### 4.3 Kingdom Animalia

Remains of animal origin were observed in low numbers across the population and they were grouped in two taxonomic categories: invertebrates and vertebrates. Due to the scattered nature of the remains from this kingdom, we have provided the skeleton numbers of their occurrence and reported only wool in Table 1.

*Invertebrates* were more abundant and consisted mainly of insect remains. Insect parts were observed in the calculus of five individuals, SK 4.26 (female) retained an insect part (probable appendage, Fig. 3 D), an unsexed individual (SK 2.11) had a well-preserved nematode (Fig. 3 E), and three individuals (two males, SKs 5.16 and 5.29, one female SK 62.02) presented with nearly complete insect exoskeletons potentially from mites (*Acaris* spp.) in their calculus (Fig. 3 F). Insects and nematodes are almost ubiquitous in the soil and mites are common food contaminants; both may indicate lack of food hygiene (Baker, 2009; Hall and Kenward, 1989; Juhola et al., 2019).

*Vertebrates* were represented by low numbers of mammal hairs and some fragments of feather barbules (Fig. 3 G). Wool (*Ovis aries*, mammal hair) was identified, as were other mammal hairs, by the ‘scales’ or keratin cuticle which covers the hair shaft (Petraco and Kubic, 2003) and was observed in only one individual (SK 2.37, male). In addition to the identified wool fibre, two individuals (SK 3.44, unsexed and SK 5.16, male) retained mammal hairs that could not be identified, as the necessary characteristics were not fully visible. Although not identified to species level, these fibres may indicate interaction with animals or the wearing of fur clothing.

### 4.4 Kingdom Fungi

Fungal spores, hyphae and yeast spores have been reported in dental calculus samples from a number of studies, often in very low numbers, where they are difficult types of remains to be taxonomically identified (e.g., Buckley et al., 2014; Power et al., 2015; Radini et al., 2017). As noted by Radini (2016), the identification of fungal remains to species level requires a well-trained analyst with a solid background in mycology and with a large purpose-built reference collection, as well as knowledge of the type substrate or environment the fungal material has likely grown (Li et al., 2007, 98). Naturally, this is not often possible in archaeological material, and often biomolecular methodologies are the only means to achieve secure species identification. Finally, most identifications of fungal remains based on spores rely on prior knowledge of the origin of the samples, such as indoor rooms (e.g., bathroom floor/ceiling; kitchen floor), outdoor settings (e.g., soil, wood, crops), with a number well documented of parameters affecting their growth (such as temperature, humidity, light, nature of the surface). In this study the number of remains attributed to the kingdom Fungi was astonishingly high (n = 594, Table 1) compared to dental calculus studies from previous periods, and will be considered in further detail below. Remains from this kingdom were recorded in 28 of 36 (78%) individuals (12 males, 11 females and 5 unsexed), however, secure species identification in the absence of culturing or DNA analysis is nearly impossible, when coupled with the lack of information of the substrate of origin (Wu, 2007 105). In this study we have encountered potential fungal plant pathogens, such as *Alternaria-* and *Torula-*types, as well as *Spegazzinia/Ulacladum* type (Fig. 3 H) which are most often associated with damp-rot of wood and cereals. These fungal types are abundant in the air and cause a variety of allergic conditions ([Bibr bib45][Bibr bib55]; Levetin, 2004; Navi et al., 1999; Wang et al., 2017) but the majority of fungal remains will need a specialised reference collection and biomolecular techniques for their identification. Yeast spores were observed in 14 of 36 (39%) individuals (9 males, 8 females, 3 unsexed). Yeast spores are treated together in this survey since they cannot be identified to species level as many species have the same visual characteristics, such as a visible budding scar (Fig. 3 I). There are at least two common potential pathways of yeast inclusion: through food and drink (e.g., *Saccharomyces cerevisiae*) or through their presence as members of the oral microbiome (*Candida albicans*) (Järvensivu et al., 2004; Lin and Heitman, 2005). Fungal hyphae were also found. Due to lack of secure identification, fungal spores were counted according to two separate groups: yeast-like fungi and non-yeast fungal remains.

### 4.5 Burnt debris

This category of debris was the most sparsely observed in the Cross Street population, with only six individuals displaying soil or mineral grit and four with burnt material or fine soot-like material. Following previous work (Hardy et al., 2016), in this study we consider particles of microcharcoal as microscopic fragments of charcoal below 180 μm (Blackford, 2000), which can be securely identified using light microscopy when the structural features necessary for conclusive identification are clearly visible (Rhodes, 1998). The burnt debris retrieved in this study consisted of small deposits of fine soot-like material incorporated into the dental calculus (3 males, 3 females, Fig. 3 J) which were too small to be identified. Nevertheless, a range of inorganic particles below 3 μm found in soil can be potentially confused with microcharcoal under light microscopy, appearing brown to black, opaque, with sharp angular edges (Blackford, 2000; Petraco and Kubic, 2003; Rhodes, 1998). Finally, the lack of clear evidence of microcharcoal found in this study will be discussed below, but must not be taken as evidence for the lack of exposure to smoke and the use of fire.

### 4.6 Other remains

The incorporation of soil and mineral grit into dental calculus was sparse in this population (1 male, 4 females, 1 unsexed). A variety of dental calculus flecks appeared stained with ‘colour’ that could be the results of bleeding of the gum, the use of food colourants, or a combination of both. Such stains could therefore be the result of food colouring practice that was common in the Georgian period (e.g., Kellet et al., 1780; Farley, 1792), however, light microscopy alone is insufficient to address the origin of such stains, and work is currently in progress to overcome these limitations.

## 5 Discussion

The variety and quantity of remains retrieved in this study can be viewed from multiple stand points, with important implications for the archaeological study of the Industrial period (ca. 1750–1850) as well as dental calculus research more generally. Overall, the typologies of the remains retrieved in this study, from starch granules to fungal spores, have been found previously in dental calculus research across different periods of time and locations (e.g., Warinner et al., 2014; Jovanović et al., 2021; Tromp et al., 2020). Nevertheless, we observed striking differences from the Medieval to the Post-Medieval period in both the quantities of microdebris, as well as the size and preservation quality of the remains, including the poor preservation of many inclusions, the high concentration of fungal spores, and the presence of exotic plant species.

Here we discuss the implications of our results from multiple perspectives. First, we discuss the implication of the findings with reference to *deliberate consumption* of foods, including their implications for our understanding of food access, as well as production and processing, and storage of food in the Post-Medieval period. Second, we then discuss the evidence that cannot be securely linked to deliberate consumption of food, with a focus on food hygiene. Other typologies of debris not linked to food consumptions are then considered, such as fibres and respiratory irritants, and their implications for health and the lived experience in urban environments. We then briefly compare our results with previously published studies from preceding time periods to better contextualize our dataset temporally. We conclude our discussion with methodological implications for future dental calculus studies focused on this fascinating historical period.

### 5.1 Food access, processing and storage

In terms of microdebris of dietary origin, starch granules have been the focus of dental calculus research from the very early stages of the discipline, and they are predominant in dental calculus research (e.g., Charlier et al., 2010; Radini et al., 2017). However, our study demonstrates two important and novel insights. First, although starch granules were recorded throughout the course of this study, many were heavily damaged, precluding taxonomic identification. Moreover, many starches were bloated and cracked, a fact that can be interpreted as the result of prolonged heating with and without water (Henry et al., 2009). Such damage was ubiquitous despite the very recent temporal origin of the remains, therefore excluding time-dependent diagenetic processes as the origin of such alteration. Thus, we hypothesize that this high level of damage is a direct consequence of more extensive food processing during this period, which would have exposed more processed plant material to prolonged periods of boiling in metal pots and food preserving, as well as technical innovations for milling finer, whiter flour cheaply with new methods for separating the elements of wheat kernels such as bran (Tann and Jones, 1996). Further evidence in support of this interpretation is the extensive fragmentation of most of the plant remains, which also lacked diagnostic features due to damage, most likely caused by food processing techniques. The poor state of preservation of starch granules and small fragmentation of plant tissues —likely belonging to leafy greens— has been noted in two additional published studies for the Post-Medieval period. Millard et al. (2020) noted smaller sized particles in the dental calculus remains of the Scottish soldiers from the Battle of Dunbar 1650, during the English Civil War (1642–1651), as well as the poor preservation of starch. Similar observations were made by Bleasdale et al. (2019) in their first multi-tissue study of diet in seventeenth century London. The authors stressed the very small size of particulate matter, preventing identifications of the plant remains, and likewise reported heavily damaged starch, with identifications limited to Triticeae (wheat and barley) and *Avena* spp. (oats). Our hypothesis is that the large quantity of undiagnostic plant remains and damaged starch granules which appear in these Post-Medieval populations are the result of heavily processed/cooked food, also supported by ample historic evidence of cooking techniques, such as lengthy boiling, sieving and mashing of various foods with plenty of ‘mock’ recipes (i.e., cucumber for mango pickles) in period cookbooks such as *Modern Domestic Cookery* by Elizabeth Hammond (1816) and *The Art of Cookery Made Plain and Easy* (1747) by Hannah Glasse (1747). These recipes were heavily marketed to middle-class women and their servants (rather than the upper-class) from the 1730s onward (Bickham, 2008, 97). Methods for pickling and preserving require boiling foods in sugar over several days with the addition of ‘greening’ (copper pennies and alum) (Kellet et al., 1780, 125–44). Later cookery books, however, were full of warnings against ‘greening’ and cautions about bread flour adulterated with bean-meal, chalk and bone-meal (Farley, 1792, 432–8). Milling techniques were also improved and finer flours were ground, removing all but the whitest portion of the flour (Sharrer, 1982). For example, steam milling, combined with mechanized shelling, bolting and sieving, produced finer flour with fewer impurities (Cullen, 1977; Trinder, 1993). This ‘purification’ process could also explain the lack of other debris such as phytoliths and cereal bran in Post-Medieval dental calculus, which have been observed in studies focused on earlier time periods, for example in Medieval England (Radini et al., 2016). Furthermore, pre-milled flour was routinely purchased from grocers, increasing the potential for spoilage and adulteration with non-wheat flours throughout the eighteenth century in English cities (Pelizzon, 2000, 39). Our observations of microdebris reflective of a more refined and processed diet also matches with the relatively poor dental health observed in the skeletal assemblage as a whole, with high prevalence of tooth decay, abscesses, and ante-mortem tooth loss, consistent with a diet high in sugars and processed carbohydrates (Keefe and Holst, 2017).

In our study, the few starch granules that could be identified belonged to staple foods from the period such as oats, legumes, and cereals of the Triticeae tribe (barley and wheat). Remains of these food crops are routinely retrieved in dental calculus studies. Among the identifiable species, two were found to be New World crops: corn (*maize)* and tapioca*,* the starch extracted from the cassava root, which are well known morphologically in starch research (Torrence and Barton, 2016). Maize can grow well in Northern Europe if sown later in Spring, but tapioca was very likely an imported plant species. The first mention of tapioca starch in England appears to be *The Gentleman's Magazine*’ v58, Part 2 - Page 721 that dates to 1788, and later appears in cookery books. Starch from tapioca is extracted from the tuber and descriptions for the correct identification of its starch morphology appear consistently in microscopy books aimed to detect food adulterations in the nineteenth and early twentieth centuries (e.g., Hassall, 1855; Greenish, 1910; Winton and Moeller, 1906), suggesting tapioca was economically important at the time. Although previously detected in Caribbean populations (Mickleburgh and Pagán-Jiménez, 2012), to our knowledge this is the first evidence of tapioca starch in dental calculus in England. The presence of these species is a direct consequence of increased long-distance trade and the general interest of Georgian populations for exotic novelties. The presence of exotic species of plants from the New World in this period was also noted by Geber et al. (2019) in nineteenth century individuals from Ireland: their study provides direct evidence of the dependency on relief food (maize) and staple foods of the poor (potato) in Ireland around the time of the Great Famine (1845–1852). Our study provides further evidence of the inclusion of maize in the diet of the English urban middle-class and complements this with the consumption of tapioca. Moffett (2006, 55) stressed the lack of synthetic work regarding different plant foodstuffs in their archaeological and social context, and notes that it is difficult to understand who could access which foods based on the traditional archaeobotanical record alone. Our study confirms that dental calculus can provide novel evidence for the distribution of certain types of plant food per capita, between sexes, and across time and space, depending on the available study populations.

### 5.2 Non-dietary evidence and food hygiene

Our study identified fungal and yeast remains as the second most abundant inclusions in dental calculus from the Post-Medieval period in England, and in particular, we note a great variety of fungal spores. Fungi include species that are edible (i.e., mushrooms, brewer's yeasts) but also species of plant pathogens and indoor microorganisms responsible for mould and food spoilage (Li et al., 2007), whereas several species can act as opportunistic pathogens in humans (Rippon, 1982). While it is not possible to achieve secure taxonomic identification for all of these fungal remains, the sheer abundance of fungal particles and spores is an important observation for this period. While many studies in dental calculus only report plant remains such as starch and phytoliths, making comparisons difficult (Radini et al., 2017), in those studies where fungal remains were reported (e.g., Warinner et al., 2014; Hardy et al., 2016; Power et al., 2015; Radini et al., 2016) these appear in much lower numbers than observed here. Fungal spores, hyphae and yeast are ubiquitous in soil and represent a serious risk of contamination or intrusions from the burial environment. The systematic contamination controls implemented in this and previous studies (Warinner et al., 2014; Radini, 2016), combined with evidence of fungal spores embedded within the calculus matrix, confirms the fungi observed in this study are not a result of laboratory or soil contamination. The significant quantity of fungal remains may result from several factors, including changes in housing conditions (which differed significantly between middle and working-classes), food storage, and food hygiene, which could be the due to a combination of fungal spores naturally present in the environment where they can accumulate on unclean surfaces in damp conditions, for example, mould on food, wood or other organic surfaces (Singh et al., 2010). Additionally, the high number of fungal spores and hyphae may be due to changes in the way food was transported and stored, as well as increased food adulterations and poor food hygiene known for the period. Longer distance transport around England, for example, during the eighteenth and early nineteenth centuries may have created the conditions for accumulation of spores. It is known that urban populations were heavily reliant on foodstuffs being brought in from the surrounding countryside, and as transportation gradually became cheaper and faster with advent of direct river and canal access as well as the turnpike road system (Bogart, 2017), it seems likely that opportunities were taken to sell more easily transported produce such as cereal staples further afield. Manchester was known as a distribution hub for imported food and goods with a multitude of warehouses and transport networks via roads and canals from Liverpool to London and the surrounding areas (Kidd, 2002, 17 & 35). Oats and wheat were brought in from Wales and the southeast of England for distribution throughout the north, in addition to meat, dairy products and market vegetables from Lancashire farms (Scold, 1992).

Expanded international shipping networks to the United States of America also facilitated food imports, such as maize, for which starch was recorded in the Cross Street population. It is possible that an increase in the number and prevalence of fungal remains throughout this population indicates that long-term storage and transport of cereals was a growing problem for the city as a whole, including middle-class consumers. We hypothesize that changes in food processing technologies, food storage methods and longer-distance transport of processed goods are being reflected in smaller overall particle size, increased fungal spores and species-specific fungal pathogens. We stress the need for future studies, in conjunction with in depth historical evidence and more extensive fungal reference collections, to explicitly test this hypothesis.

### 5.3 Non-dietary evidence: fibres and potential respiratory irritants

This section focuses on the microremains that could result from dust in the environment —i.e., that are not deliberately ingested— and that can be respiratory irritants if inhaled in large quantities, particularly textile fibres, wood and burnt debris. Textile fibres identified as bast fibres such as flax/hemp were distributed equally between sexes in the Cross Street population sample and in similar quantities, suggesting they were part of the lived environment. Flax and hemp were common fibres in a variety of textile products, including sacks, clothes and a variety of house linens. Particularly interesting is the presence of cotton in our assemblage, which is likely linked to the ubiquity of cotton as a raw material due to the role Manchester played in cotton textile manufacturing (Kay-Shuttleworth, 1970; Collier, 1964). Furthermore, both maize and cassava or tapioca starch were commercially important in the treatment of cotton fabrics (Winton and Moeller, 1906, 664–5), which may relate to cotton processing in Manchester at the time; however, it should be noted that those individuals with tapioca in their dental calculus do not have concurrent evidence of cotton. Additionally, the cloth finishing industry in the eighteenth century primarily used wheat and potato starch rather than that of maize or tapioca and therefore it is more likely that the latter were incorporated into the dental calculus via dietary elements rather than textile production (Peckham, 1986).

The widespread presence of wood-dust debris —a known respiratory irritant when present in high concentrations— across the population (64% of the assemblage) has proved problematic in that there is no way to determine its origin. Wood products and wooden building material were widely used in the Post-Medieval period, and perhaps in times of housing expansion, as in the city's rapid expansion in the late eighteenth century (Nevell, 2011), may have been easily inhaled through the air. Wood-dust however was widely used as an adulterant in medicine and food (particularly flour), and thus both modes of inclusion (inhalation and ingestion) cannot be excluded (Winton and Moeller, 1906). Further studies are required to contextualize this finding in additional Post-Medieval populations from different socio-economic and environmental (e.g., urban vs rural) contexts.

Particularly interesting is the low frequency and quantities of microcharcoal and burnt debris retrieved from this population, especially when contrasted with the dental calculus record from earlier populations and considering the historical evidence for high smoke exposure in the region at the time (see below). Microcharcoal and burnt debris can enter the mouth by adhering to cooked food or through accidental inhalation, and is routinely found in dental calculus (Radini et al., 2017). The number and modalities of inclusion of burnt debris in calculus makes it difficult to interpret (Radini, 2016). Here the lack of evidence for microcharcoal and burnt debris is also difficult to address —as this cannot be considered evidence of absence of an exposure to smoke or cooked/roasted food— some consideration can be made in regards to the nature of the fuel used by this population and how this may affect its visibility in dental calculus. It is well known that the use of coal in London and later throughout England increased widely during the Later Medieval period and became a real issue of air pollution in the Victorian Era (Bowler and Brimblecombe, 2000; Fowler et al., 2020). The use of coal as fuel generates a very fine particulate matter that may not even be visible by light microscopy and therefore may not leave any trace in calculus, unless with frequent close exposure to the source of the fuel. The interaction of coal soot with the saliva and its clearance from the oral cavity is not yet fully understood. Our study therefore highlights the need for additional research into the visibility of exposure to coal smoke in calculus, as well as the need for a better understanding of burnt debris in dental calculus more generally.

Finally, while fungal spores have implications for food processing, storage and transport, they also have important implications for respiratory health (Sorenson, 1999) as spores and moulds can be considered respiratory irritants, especially in large quantities. These spores and hyphae, combined with (potentially inhaled) wood dust, could represent a significant increase in particulate matter and respiratory irritants experienced during this period. Exposure to high levels of particulate matter can affect both the upper and lower respiratory tract. In the lower respiratory tract (trachea and lungs), air pollutants can be implicated in cancer, bronchitis, emphysema, asthma and various allergies. Dust reaching the lungs can result either in a simple accumulation, or generate a reaction in the lung tissues that may be severe enough to impair lung function, such as byssinosis (caused by cotton and flax dust), or cause fibrotic lung diseases, such as silicosis (caused by free crystalline silica dust). On the other hand, inhalation of high levels of smoke and soot causes anthracosis (Amoli, 1998). The upper tract of the respiratory system (nose, maxilla, sphenoid, ethmoid, and frontal sinuses) is often affected, by air pollution, and one of the most common conditions is maxillary sinusitis, an inflammation of the paranasal sinuses that causes severe discomfort and alteration of the soft and bony tissues of the sinuses (Slavin et al., 2005); in skeletons, chronic maxillary sinusitis is characterized by pitting or spicular bone formation on the internal surfaces of the maxillary sinuses (Roberts, 2007). Maxillary sinusitis is a complex pathology, which may result from upper respiratory tract infections, pollution, smoke, dust, allergies, as well as dental abscesses which penetrate the sinus cavity (Roberts and Manchester, 2005, 174–176; Collins, 2019) and produces skeletal lesions in 60% of modern patients (Boocock et al., 1995, 484). Skeletal populations from both urban and rural sites often have very high prevalence rates of maxillary sinusitis, with males and females being equally affected irrespective of dental disease (Roberts, 2007, 792; Panhuysen et al., 1997). Studies have linked this pathology to polluted environments in urban dwellers of different time periods, including the Post-Medieval period (Roberts 2007, 2020). Recording the true prevalence of chronic maxillary sinusitis in the Cross Street Cemetery assemblage was undertaken where possible, but was hampered by the number of intact crania, for which the sinus cavities could not be observed (Keefe and Holst, 2017). In our study, three individuals affected by this pathology also had high levels of fungal spores in their dental calculus (SK2.36, SK5.16, SK4.36, Supplementary Table S1). Three other skeletons (SK1.37, SK2.31 and SK5.29) also had relatively high levels of fungal spores, but their sinuses were either intact or absent, and thus we were unable to assess this pathology. Only one skeleton (SK2.15) had high fungal spore counts but no evidence of maxillary sinusitis. While it is not possible to confirm that high exposure to fungal debris is the direct cause of maxillary sinusitis in these affected individuals, it is possible that high concentration of spores in the environments may be a contributing factor. Systematic studies testing the correlation between respiratory irritants and maxillary sinusitis will be needed to understand how particulate matter in calculus can contribute to our understanding of respiratory health and urbanisation (Radini, 2016). Such studies however will need to take in account the numerous pathways of debris inclusion in calculus as a complicating factor.

### 5.4 Brief comparison with preceding periods

In this section we provide a brief comparison with selected data sets from Medieval and earlier periods, focusing specifically on the issue of preservation and quantity of remains. This comparison, however, deliberately excludes comparisons with calculus from Neanderthal or other hominins from the deep human evolutionary past, as the status of preservation may be heavily influenced by diagenetic processes (Collins and Copeland, 2011), and the integrity of starch granules found in calculus for such ancient material is currently under debate (Copeland and Hardy, 2018). With relation to starch, even a brief comparison with the preservation of starch granules from earlier data sets, provides further evidence that the poor status of preservation of our cereal starch granules was due to heavy processing of raw materials known in the eighteenth and nineteenth centuries. Intact amyloplasts still retaining starch granules, were recovered by Cristiani et al. (2016) from the calculus of Mesolithic foragers in the Balkans and also by Buckley et al. (2014) in pre-agrarian Sudan. Experimental work performed by Zupancich et al. (2019) in grinding cereals with stone implements continued to preserve starch amyloplasts in the resulting flour. Well preserved starch granules were also found in prehistoric (e.g., Henry et al., 2011; Li et al., 2010; Cristiani et al., 2016) and historic populations from the Roman (e.g., D'Agostino et al., 2019) to the Later Medieval Periods (e.g., Radini et al., 2016). A further example of this is the evidence from Medieval Italian human dental calculus reported by Gismondi et al. (2018) who describe excellent preservation of easily identifiable starch granules with abundant phytoliths, plant fibres and pollen, indicating that these individuals were much closer to food processing (cereals in particular) than were those living in industrial urban settings, who were more likely to purchase ready-milled flour or bread rather than grinding grain at home. In striking contrast, individuals from our study display no evidence for plant phytoliths or pollen debris. There is therefore a record of well-preserved starch granules in pre-agrarian and agrarian societies thousands of years old which precludes any suggestion of diagenetic changes in Post-Medieval starch granule recovery from human dental calculus. A comparison with preceding periods therefore suggests that the poor preservation of starch granules in this study is the result of processing and cooking practices, as well as decreased exposure to the actual processing of starchy products from raw materials before consumption (e.g., milling).

The other important comparison is in relation to the quantity of small particulate matter of plant origin and fungal spores in this study. The sheer quantity of remains observed in our population (n = 9389 particles) is particularly striking when the starting sample size is considered, often below 1 mg of calculus. Despite the relatively small quantities of calculus, over 500 fungal remains were identified across the data set, with a mean of 247 for males, and 334 for females. In contrast, a variety of studies with similarly small samples weights displayed a very small quantity of identified remains, and in particular fungal remains, when compared with ours: Buckley et al. (2014), for example, found one single spore from a sample of less than 0.5 mg in weight; Power et al. (2015) retrieved 37 fungal spores from a sample of 1.4 mg in weight. Fungal remains were nearly ubiquitous in the study by Radini (2016), but usually in single counts for samples often >20 mg in weight. Juhola et al. (2019) in their study of Iron Age individuals in Finland connected the small sample size of calculus with the low amount of material retrieved from the analysis. Our work suggests that while dental calculus size likely has an effect on the material captured by its matrix, the typologies and size of remains entering the mouth may also play an important role.

### 5.5 Methodological implications and future directions

The study highlights the value of dental calculus to ‘capture’ a variety of information on past populations, we can summarize them in the following key points:

1. There is increasing evidence that dental calculus can record the arrival of imported species at the individual and at the population level. In order to maximise our understanding of the arrival and distribution of novel plant and animal products, we encourage dental calculus researchers to contribute to creating a wider and more accessible reference collection of plant and animal microremains that reflects cultural movements of people and goods through space.
2. The starting weight of the dental calculus sample may not be fully responsible for the quantity of remains that can be observed via light microscopy. Instead, size of the ingested or inhaled particles may also influence what information dental calculus can capture, as demonstrated by the large number of very small particles that became entombed in the small calculus samples within this study.
3. The preservation status of starch granules in dental calculus could provide important information on the nature of the processing and cooking practices even for relatively recent populations, as well as insight into food transport and storage. Our study provides further confirmation that poor preservation may not just be the result of the age of the starch itself or diagenetic processes.
4. Fungal remains, often grouped in the category of non-pollen palynomorphs, have mainly been recovered from archaeological sediments and coprolites, and have largely been a neglected line of evidence (Haas, 2010). While fungal remains have been identified in the dental calculus of some populations, understanding the factors influencing fungal quantities and diversity will not be possible until fungal remains can be taxonomically identified more securely than is possible using current comparative methodologies. This study, combined with those where evidence of fungal spores and hyphae was reported, clearly suggest that dental calculus is a reservoir of this neglected evidence. Future work incorporating more comprehensive reference collections and novel biomolecular methodologies are imperative for unlocking this potentially very valuable source of information on past diet, subsistence, health and environment.
5. There is a clear need for enhancing our understanding of how exposure to smoke from different fuel sources can be identified and quantified in dental calculus. It is possible that chemical markers, as attested by Hardy et al. (2016) may allow us to investigate the economic and health impacts associated with the shift from wood to coal from the later Medieval period.
6. Dental calculus may provide novel insight into textile fibres (from both plants and animals) including their introduction, diversity, and use; we encourage dental calculus researchers to record and report this line of evidence in future studies.

Our study confirms the utility and importance of light microscopy and dental calculus analyses in Post-Medieval bioarchaeological research, and highlights the novel insights that may be gleaned from this approach even when starting quantities of calculus are limited in size. Here, we focused on a middle-class, urban, Industrial Period population, uncovering a number of key observations which should be systematically evaluated across space and time, and within populations from different socio-economic classes. Nevertheless, since many dental calculus studies only report starch and phytoliths, our ability to compare our novel findings to previous time periods is limited (Radini et al., 2017). Due to the relatively small quantities of dental calculus that may be present within many archaeological assemblages and the variety of remains that can be retrieved, it is paramount that all classes of micro-debris are recorded and reported in future studies, even when it is not possible to securely identify them taxonomically. Our study in fact proves that even the quantity of one category of remains, such as Fungi, and status of preservation alone can provide useful insights into past life if comparative data with other populations are available.

## 6 Conclusions

Using light microscopy, our study analysed microdebris entrapped within the dental calculus of an assemblage of Industrial period, urban, middle-class individuals from Cross Street Chapel cemetery, Manchester, UK. We identified a range of microremains, including starches and other plant tissues, fungal and yeast spores, wood particles, insect remains, plant and animal fibres, and burnt material. In particular, we noted an extremely small particle size for most plant tissues, precluding taxonomic identification, with starches that were heavily damaged, consistent with extensive processing and cooking. In comparison with previous time periods, this extensive damage and fragmentation of plant microremains (combined with the conspicuous lack of plant phytoliths) are consistent with more extensive food processing techniques developed in the Industrial period. In addition to Old World staples of Triticeae (wheat, barely), legumes, and oats, we found evidence of New World foods, such as maize and tapioca starch, confirming the ingestion of these imported foods by urban, middle-class individuals. Compared to previous time periods, we also found an exceptionally high number of fungal and yeast spores, potentially signalling changes in food storage, transport as well as changes in housing and living conditions. Interestingly, we found little evidence for burnt debris and microcharcoal, which may be related to differential preservation of fine particulates associated with the use of coal as a fuel after the Late Medieval period. Our study raises several new hypotheses about how the process of Industrialisation, particularly in terms of diet, hygiene and air quality, was experienced by middle-class, urban dwellers in the late eighteenth and early nineteenth centuries. Further systematic analysis of Post-Medieval populations from a range of geographic and temporal contexts are required to expand our understanding of how urbanisation and industrialisation was experienced by individuals of different social classes and in urban and rural environments.

This investigation systematically recorded and reported all micro-debris observed in human calculus using light microscopy (see Blatt et al., 2011; Radini et al., 2017) and presents insight into a time period which has been relatively understudied through dental calculus analysis (Bleasdale et al., 2019; Geber et al., 2019). The analysis conducted here, solely with optical microscopy, shows the potential of microscopic particles entrapped in the dental calculus matrix to contribute a wealth of information about diet and the lived environment on both an individual and populational level during the Industrial period, and Post-Medieval period more generally. The study shows that a variety of remains in calculus need further investigation. In particular, more research is required to better identify and interpret fungal remains, detect exposure to a variety of air pollutants including smoke, and document the arrival and distribution of imported plant species. Finally, additional studies incorporating individuals from a greater range of social classes and environments (e.g., urban, rural) from Post-Medieval England are essential for providing more nuanced insight into how changes in food processing, storage, hygiene and respiratory health were experienced across this dynamic period in history.

## Author contributions

Lisa MacKenzie: Conceptualization, Methodology, Investigation, Writing - Original Draft, Camilla Speller: Conceptualization, Supervision, Writing - Review & Editing Malin Holst: Investigation, Resources, Writing - Review & Editing Katie Keefe: Investigation, Resources, Writing - Review & Editing Anita Radini: Methodology, Validation, Investigation, Writing - Original Draft, Writing - Review & Editing, Supervision.

## Data availability

All data is available within the manuscript or supplementary material tables.

## Declaration of competing interest

The authors declare that they have no known competing financial interests or personal relationships that could have appeared to influence the work reported in this paper.
